# The accuracy of blood pressure measured by arterial line and non-invasive cuff in critically ill children

**DOI:** 10.1186/s13054-016-1354-x

**Published:** 2016-06-08

**Authors:** Rachel Joffe, Jonathan Duff, Gonzalo Garcia Guerra, Jodie Pugh, Ari R. Joffe

**Affiliations:** University of Alberta, Faculty of Science, Edmonton, Alberta Canada; Department of Pediatrics, University of Alberta and Stollery Children’s Hospital, Edmonton, Alberta Canada; 4-546 Edmonton Clinic Health Academy, 11405 87 Avenue, Edmonton, Alberta T6G 1C9 Canada

**Keywords:** Arterial line, Blood pressure, Children, Intensive care units, Pediatric, Monitoring, Physiologic

## Abstract

**Background:**

The accuracy of arterial lines (AL) using the flush test or stopcock test has not been described in children, nor has the difference between invasive arterial blood pressure (IABP) versus non-invasive cuff (NIBP) blood pressure.

**Methods:**

After ethics approval and consent, we performed the flush test and stopcock test on AL (to determine over damping, under damping, and optimal damping), and determined the difference (NIBP–IABP) in systolic, diastolic, and mean blood pressure (ΔSBP, ΔDBP, and ΔMAP). The primary outcome was incidence (95 % CI) of optimally damped AL. Predictors of ΔBP (effect size (95 % CI)) were determined using multiple linear regression.

**Results:**

There were 147 AL tests in 100 enrolled patients with mean age 44.7 (SD 56) months, weight 16.8 (SD 18.3) kg, male 59 %, postoperative-cardiovascular 52 %, peripheral-AL 78 %, inotropes 29 %, vasodilators 15 %, and ventilated 73 %. The flush test performed in 66 patients (45 %) showed optimal damping in 30 (46 %; 95 % CI 34, 57 %), over damping in 25 (38 %) and under damping in 11 patients (17 %). The stopcock test was over-damped in 128/146 patients (88 %), with the same damping as the flush test in 24/64 (38 %). In optimally damped (flush test) AL, ΔSBP, ΔDBP, and ΔMAP were 0.8 (SD 12.2), −5.2 (SD 8.7), and −4.9 (7.6) respectively. A second set of AL tests was done 2 h later on the same day in 62 patients; AL damping often changed (10/28 flush tests) and ΔBPs correlated poorly (*r* = 0.31–0.55). Predictors (effect size) of ΔDBP were vasodilator infusion (15.6 (2.9 to 28.3); *p* = 0.016) and optimal damping (−7.2 (−12.2 to 2.2); *p* = 0.005); and of ΔMAP were vasodilator infusion (10.0 (−0.3 to 20.4); *p* = 0.057) and optimal damping (−4.0 (−8 to 0.1); *p* = 0.058). There were no independent predictors of damping category (n = 66 flush tests).

**Conclusions:**

Optimally damped AL occur in half of critically ill children, and this is not predictable. There is much variability in ∆BP between NIBP and the gold standard IABP, and this varies even in the same patient on the same day, and is not easily predictable. In critically ill children, NIBP may not be accurate enough to guide management, and more attention to ensuring the AL is optimally damped is needed.

**Electronic supplementary material:**

The online version of this article (doi:10.1186/s13054-016-1354-x) contains supplementary material, which is available to authorized users.

## Background

Blood pressure is a crucial vital sign in critically ill children. Accurate measurement of blood pressure is assumed in the diagnosis of hypovolemic, cardiogenic, vasodilatory, and obstructive shock, and of hypertension from any cause. Accurate measurement of blood pressure is also assumed in the (often urgent) management of any of these conditions with volume, vasoactive medication infusions, and even extracorporeal support. Even triage and resource allocation decisions, such as whether to transport and admit to the pediatric intensive care unit (PICU), are often based on the assumption of accurate blood pressure measurement. Nevertheless, there is no study we are aware of that determines the accuracy of blood pressure measurement in children in the PICU, whether invasive arterial blood pressure (IABP) is measured using an invasive arterial line (AL), or whether non-invasive cuff blood pressure (NIBP) is measured.

Accurate IABP measurement requires an optimally damped measurement system, and if the pressure is over or under damped the measured IABP is theoretically underestimated or overestimated, respectively [1, 2]. Determination of the damping condition of the AL can be done using a flush test, whereby a small volume of fluid is rapidly infused into the system, and the subsequent ‘ringing’ of the waveforms is recorded and used to calculate the natural frequency (how rapidly the system oscillates after a stimulus) and amplitude ratio (or damping coefficient; how quickly the system comes to rest due to frictional forces after a stimulus) [1, 2]. This test was first described in 1981, and has since been used in studies in adults in intensive care, and is suggested in standard anesthesia texts [1–4]. This test can easily be done when AL are set up with an Intraflo continuous flush element. Alternatively, it has been suggested that closing the stopcock to the continuously infusing AL for several seconds followed by quickly opening the stopcock will result in a similar rapid flush to the system [1]. To our knowledge, the usefulness of this stopcock test has never been reported.

In this study we aimed to determine the accuracy of AL measurement of IABP using the flush test and stopcock test. In addition, we aimed to determine the difference between IABP and NIBP in critically ill children, particularly when the AL is known to be accurate (optimally damped).

## Methods

### Ethics statement

This study was approved by the Health Research Ethics Board of the University of Alberta. Although NIBP is routinely measured in the PICU, the ethics committee required signed informed consent from legal guardians prior to inclusion in the study because it was decided that the extra NIBP measurement may be of discomfort to the patients.

### Study procedures

All patients in PICU at Stollery Children’s Hospital, and who had an AL, were eligible for the study from late June to mid October 2015. Exclusion criteria were: extracorporeal life support; abnormal aortic arch, including after subclavian flap repair of coarctation; known non-functioning AL (e.g., losing waveform and no longer thought to be accurate, or unable to withdraw bloodwork); ongoing patient agitation; AL in an umbilical site; or lack of signed consent. A case report form and study instruction manual were created prior to patient recruitment, with standard definitions, calculation instructions, and procedure instructions (Additional files 1 and 2). Demographic (age, sex, diagnostic category), severity of illness (inotrope infusion score, vasodilator infusion in use, ventilation in use), potentially confounding factors for NIBP (obesity defined as over the 90^th^ percentile weight for age; severe edema in the limb used for NIBP; chronic hypertension; and obstructive airway disease), and site of the AL (peripheral or femoral) variables were recorded.

A flush test was done for children weighing ≥10 kg and the AL waveform printed for later calculation of natural frequency and amplitude ratio, and (using a published graph) determination of optimal, under, or over damping of the AL (see Additional file 3 for arterial line setup, and flush test demonstrations) [1, 2]. A stopcock test was then done and the AL waveform printed for later calculations. The flush test could only be done on patients ≥10 kg in weight because in our PICU the Intraflo continuous flush element is not used on smaller patients. Following the flush and stopcock tests, the NIBP was measured in a different limb to the one with the AL. The NIBP and IABP were recorded at the same time (i.e., the IAPB at the end of deflation of the cuff), including systolic (SBP), diastolic (DBP), and mean (MAP) pressures. If the difference in SBP was >10 mmHg, the NIBP and IABP were repeated, and the closest (in SBP) of the two measurements was recorded in the case report form. These procedures were done on the day (d) of the AL as follows: d1–3, d4–6, and d7–10 as appropriate; on d1–3 a second set of procedures was done 2–3 hours later on the same day if this occurred during working hours.

### Study materials

The arterial line was set up as follows. The intra-arterial catheter (24 G (5/8 in), 22 G (1 in), or 20 G (1-1/4 in)) was connected to a straight connector (ICU Medical Smallbore Extension Set with MicroClave^R^ clear; 7 in, 0.24 ml; San Clemente, CA, USA), a stopcock (Hi-Flo™ Smiths Medical, Brisbane, Australia), and high pressure tubing with disposable transducer (Edwards Lifesciences TruWave™ 3 cc/72 in (180 cm) pressure monitoring set). The transducer was connected by the invasive pressure cable into the blood pressure module of the Phillips IntelliVue MP70 bedside monitor. In patients weighing at least 10 kg, the transducer set was connected to the 300 mmHg pressure bag to run at 3 ml/h. In patients under 10 kg, the transducer set was connected to the Alaris pump (Attach SmartSite^R^ Burette Set, CareFusion, San Diego, CA, USA) to run at 1.5 ml/h. The pressure monitoring set has an Intraflo continuous flush element pigtail that can be pulled to allow rapid flush of the system, and this is functional when not on the Alaris pump. The NIBP was done using Phillips EasyCare cuffs of appropriate size, and connected with the NIBP pressure cable into the blood pressure module of the Phillips IntelliVue monitor.

### Statistics

The primary outcome was the proportion of AL that were non-optimally damped on the flush test, with adjusted Wald 95 % confidence intervals (CI). Assuming a similar prevalence of non-optimally damped AL as described recently in adults in intensive care (31 %), we estimated that a sample of 80 children will allow a reasonable 95 % CI of +/− 10 % [3]. Secondary outcomes planned were: difference between NIBP and IABP (ΔSBP, ΔDBP, and ΔMAP) according to the damping category of the AL, described as mean (SD) and median (IQR) difference, and with Bland-Altman plots [5]; comparison of the flush test and stopcock test, described as the proportion of tests having the same damping result; predictors of optimally damped AL using multiple logistic regression models; and predictors of the NIBP–IABP difference using multiple linear regression models. Pre-specified variables entered in the regression models were: gender, weight, inotrope in use, vasodilator in use, ventilation in use, peripheral site of the AL, day category of AL (d1–3, d4–6, or d7–10), diagnostic category of the patient (postoperative cardiovascular vs other), and optimally damped AL (for predicting NIBP–IABP). Finally, correlation between the first and second set of procedures on the same day (for the d1-3 category AL) was determined for ΔSBP, ΔDBP, and ΔMAP, and the difference between these variables on the two tests described. Sensitivity analyses were done using results from only the first testing of the AL that were in the d1–3 category.

## Results

### Description of the cohort

**Fig. 1:**
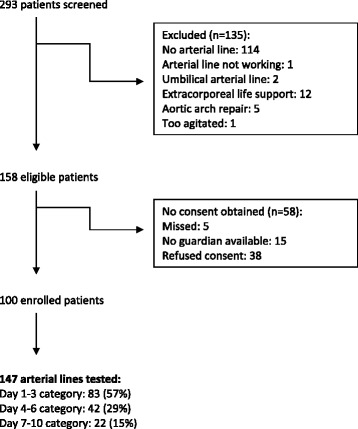
Study patient inclusion flow chart

The inclusion/exclusion patient flow is shown in Fig. 1. The 147 patients having the AL tested had mean age of 44.7 (SD 56) months, median 13.5 (IQR 4–78) months and mean weight of 16.8 (SD 18.3) kg, median 8.6 (IQR 4.8–23.4) kg, and 86 patients (59 %) were male. Diagnostic categories included postoperative cardiovascular surgery (n = 76 (52 %)); non-operative cardiovascular (n = 10 (7 %)); postoperative general surgery (n = 16 (11 %)); and medical (n = 45 (30 %)). The AL were peripheral in 114 patients (78 %: radial in 87, ulnar in 12, brachial in 8, and dorsalis pedis in 7 patients) or femoral in 33 patients (22 %). The peripheral AL were size 24 G in 12 (13 %), 22 G in 66 (73 %), and 20 G in 12 (3 %) patients. Interventions included inotropes in use in 43 (29 %) (inotrope score 6.6 (SD 3.6), range 2–18.5), vasodilator in use in 22 (15 %), and ventilation in use in 107 patients (73 %) (invasive ventilation 78, non-invasive ventilation 25, and high-flow nasal cannula in 4 patients). There were few patients with potential confounders to NIBP: obesity (n = 13 (9 %)), severe arm edema (n = 7 (5 %)), chronic hypertension (n = 6 (4 %)), and obstructive airway disease (n = 5 (3 %)).

### AL accuracy

For the primary outcome, AL was optimally damped in 30/66 flush tests (46 %; 95 % CI 34, 57 %), over damped in 25 (38 %; 95 % CI 27, 50 %), and under damped in 11 (17 %; 95 % CI 9, 28 %); thus, the prevalence of non-optimally damped AL was 36 (55 %; 95 % CI 43, 66 %). The proportions in each damping category were virtually identical in d1–3 AL and other day categories of AL (optimally damped in 45 % vs 46 %, over damped in 38 % vs 38 %, and under damped in 17 % vs 17 %). For the AL that were tested for a second time on the same day, the proportions were also similar on the second flush test (n = 29), being optimally damped in 13 (45 %), over damped in 13 (45 %), and under damped in 3 patients (10 %); however, the same damping result was obtained in only 18/28 patients (64 %). When there was ‘ringing’ of the AL (n = 41; 62 %) the natural frequency and amplitude ratio was calculated and was 22 (SD 5, median 25 (IQR 17–25)) and 0.5 (SD 0.2; median 0.5 (IQR 0.37–0.62)). The stopcock test was done in 146 patients (99 %), and demonstrated an optimally damped AL in 5 (3 %), over damped AL in 128 (88 %; always because of absent ‘ringing’), and under damped AL in 12 patients (8 %). When both were done, the stopcock test had the same result as the gold standard flush test in 24/64 (38 %). For the AL that were tested for a second time on the same day, the proportions were also similar on the second stopcock test (n = 61), being optimally damped in 2 (3 %), over damped in 56 (92 %; all with no ‘ringing’), and under damped in 3 patients (5 %), and with the same result as the second flush test in 13/29 (45 %).

### Difference in NIBP–IABP

**Fig. 2:**
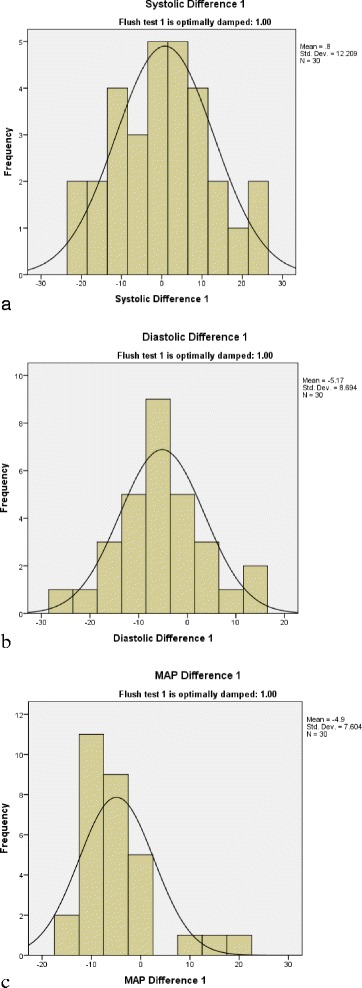
Distribution of the difference between non-invasive cuff blood pressure and intra-arterial blood pressure for optimally damped arterial lines. **a** Difference in systolic blood pressure; **b** difference in diastolic blood pressure; **c** difference in mean blood pressure (*MAP*)

**Fig. 3:**
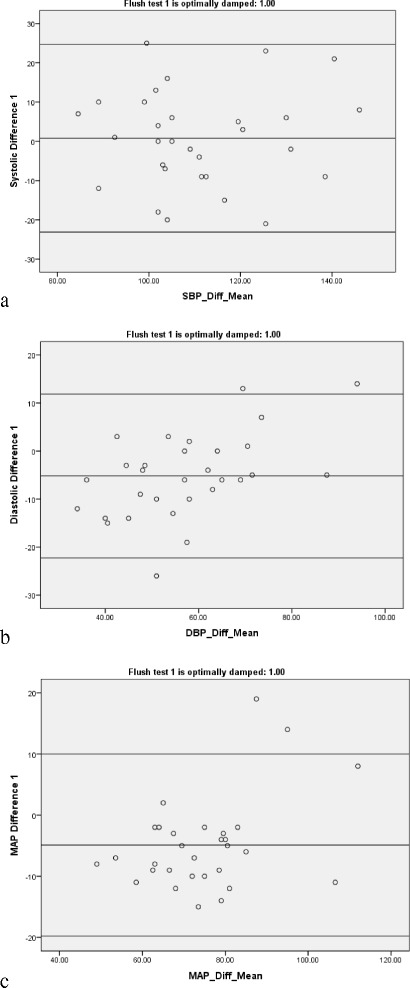
Bland-Altman plots of non-invasive blood pressure compared to invasive arterial blood pressure in optimally damped arterial lines. **a** Difference in systolic blood pressure (S*BP*); **b** difference in diastolic blood pressure (*DBP*); **c** difference in mean blood pressure (*MAP*)

**Fig. 4:**
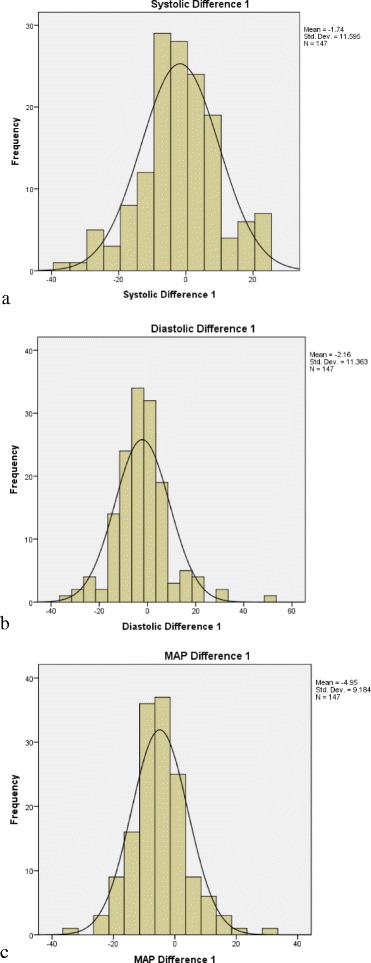
Distribution of the difference between non-invasive blood pressure and intra-arterial blood pressure for all arterial lines. **a** Difference in systolic blood pressure; **b** difference in diastolic blood pressure; **c** difference in mean blood pressure (*MAP*)

The ΔSBP, ΔDBP, and ΔMAP for each category of the AL damping on the flush test was on average small, but the SDs and IQRs were wide (Table 1, Fig. 2), and the limits of agreement on Bland-Altman plots were also wide (Fig. 3). Because the stopcock test was so often over damped due to absent ‘ringing’, and was usually different from the flush test, we did not consider determining the difference between NIBP and IABP by stopcock damping category; rather we report the results for all 147 AL tested. Again, although the mean differences were small, the SDs and IQRs were wide (Table 1, Fig. 4), and the limits of agreement on Bland-Altman plots were also wide (Additional file 4: Figure S1). The second set of AL tests on d1–3 had similar results (Table 1). Although the correlations between the same day paired (n = 62) ΔSBP (*r* = 0.55, *p* < 0.001), ΔDBP (*r* = 0.32, *p* = 0.01), and ΔMAP (*r* = 0.31, *p* = 0.013) were statistically significant, the *r* values were low, and the difference between the paired ΔSBP (0.4 (SD 12.5); median −0.5 (IQR −8.0, 8.3)), ΔDBP (−2.3 (SD 10.1); median −2.5 (IQR −8.0, 3.0)), and ΔMAP (−1.2 (SD 9.1); median −0.5 (IQR −7.3, 5.0)) were on average small, but with wide SD and IQR (Additional file 4: Figure S2).

**Table 1:** The difference between non-invasive cuff and invasive arterial blood pressure measurements

Difference NIBP–IABP	SBP	DBP	MAP
For the 66 flush-tested arterial lines*			
Optimal damping (n = 30)	0.8 (12.2); 0.5 (−9, 8.5)	−5.2 (8.7); −5.5 (−10.5, 0.3)	−4.9 (7.6); −6.5 (−10, −2)
Over damping (n = 25)	−1.5 (11.2); −1 (−7, 6)	2 (12.5); 1 (−5.5, 6.5)	−1.3 (8.5); −1.3 (−7, 2)
Under damping (n = 11)	−2.7 (12.5); −3 (−11, 6)	1.9 (8.4); 1 (−2, 7)	−1.4 (9.6); −4 (−10, 6)
For all the n = 148 arterial lines			
Combined results	−1.7 (11.6); −2 (−8, +5)	−2.1 (11.4); −3 (−8, +3)	−5.0 (9.2); −5 (−10, −1)
Combined results	*r* = 0.87	*r* = 0.78	*r* = 0.87
For the 42 flush tested arterial lines in the d1–3 category			
Optimal damping (n = 19)	−3.1 (12.2); −4 (−12, 6)	−5.4 (7.4); −6 (−10, 0)	−6.5 (5.6); −8 (−10, −3)
Over damping (n = 16)	−2.0 (12.7); −0.5 (−7.5, 5.5)	−1.9 (9.7); −1 (−6.8, 3)	−3.5 (7.8); −2.5 (−8, 0.5)
Under damping (n = 7)	−2.9 (13.8); −4 (−9, 1)	1.4 (10.4); −3 (−8, 5.5)	−2.0 (10.4); −6 (−14, −1)
For all the 83 arterial lines in the d1–3 category			
Combined results	−1.5 (11.6); −2.0 (−8, 6)	−3.5 (8.4); −4.0 (−8, 1)	−5.5 (7.0); −6.0 (−10, −1)
Combined results	*r* = 0.83	*r* = 0.84	*r* = 0.89

Given as mean (SD); median (IQR). *Analysis of variance, diastolic blood pressure (DBP) values are different by damping category (*p* = 0.024). *NIBP* non-invasive blood pressure, *IABP* invasive arterial blood pressure, *SBP* systolic blood pressure, *DBP* diastolic blood pressure, *MAP* mean arterial pressure, *r* correlation coefficient, *d* day

### Predictors of damping category

On multiple logistic regression models, there were no independent predictors of AL damping category on the flush test (n = 66). When the inotrope score was used instead of inotrope in use, there were still no independent predictors. Age was collinear with weight (*r* = 0.91); however, age was not a predictor on univariate regression.

### Predictors of NIBP–IABP

Independent predictors of ΔSBP, ΔDBP, and ΔMAP are shown in Table 2, both for AL with the flush test (with damping category entered as a variable), and for all AL (damping category not entered as a variable). Having a vasodilator in use resulted in the NIBP overestimating DBP and MAP. The NIBP underestimated DBP and MAP in patients with an optimally damped AL.

### Sensitivity analyses

The results for the first test on the AL in the d1–3 category were analyzed separately. For the 42 flush tests, the AL was optimally damped in 19 (45 %), over damped in 16 (38 %), and under damped in 7 patients (17 %). The NIBP–IABP differences according to damping category, and for all AL, are given in Table 1. There were no independent predictors of AL damping category on multiple logistic regression. Predictors of NIBP–IABP difference for flush-tested AL, and for all AL, are given in Table 2. The results of these analyses were similar to analysis including all day categories of AL.

**Table 2:** Independent predictors of the difference between non-invasive cuff and invasive arterial blood pressure by multivariable linear regression

Outcome	Variable	Effect size (95 % CI)	*P* value
For the n = 66 flush-tested arterial lines			
∆SBP	Day category	4.9 (0.2, 9.7)	0.043
∆DBP	Vasodilator in use	15.6 (2.9, 28.3)	0.016
	Day category	5.5 (1.6, 9.5)	0.007
	Optimally damped	−7.2 (−12.2, −2.2)	0.005
∆MAP	Vasodilator in use	10.0 (−0.3, 20.4)	0.057
	Day category	4.5 (1.3, 7.7)	0.007
	Optimally damped	−4.0 (−8.0, 0.1)	0.058
For all 147 arterial lines			
∆SBP	Inotrope in use	−5.75 (−11.3, −0.18)	0.043
	Peripheral AL site	5.03 (0.06, 10.0)	0.048
∆DBP	-	-	All ns
∆MAP	-	-	All ns
For the 42 flush-tested arterial lines in the d1–3 category			
∆SBP	-	-	All ns
∆DBP	-	-	All ns
∆MAP	Vasodilator in use	17.8 (1.6, 34.1)	0.032
	Optimally damped	−5.4 (−10.3, −0.4)	0.035
For all 83 arterial lines tested in the d1–3 category			
∆SBP	Inotrope in use	−8.1 (−16.1, −0.13)	0.047
	Peripheral AL site	6.3 (−0.4, 13.0)	0.065
	Weight	−0.13 (−0.27, 0.01)	0.060
∆DBP	-	-	All ns
∆MAP	-	-	All ns

*SBP* systolic blood pressure, *DBP* diastolic blood pressure, *MAP* mean arterial pressure, *AL* arterial line(s), ∆ difference (non-invasive cuff blood pressure minus invasive arterial blood pressure), *d1–3* day 1–3, *ns* not significant. There was no meaningful difference in results if inotrope score was entered instead of inotrope in use; for all arterial lines and ∆SBP the effect size for inotrope score was 0.98 (0.28, 1.68; *p* = 0.007) for 147 AL, and 1.36 (0.11, 2.61; *p* = 0.033) for all 83 d1–3-category AL

## Discussion

This is the first study we are aware of that has determined the accuracy of AL in PICU, and the first to compare NIBP to IABP in critically ill children, in whom the accuracy of the AL is known. There are several important findings from this study. First, the AL were accurate (i.e., optimally damped) in 30/66 (46 %; 95 % CI 34, 57 %) of AL that were flush-tested. In fact, the damping category of the AL could change (in 36 % of patients) even within hours on the same day. Second, the stopcock test is not a useful method to determine AL damping, as most tests (88 %) do not cause ‘ringing’, and the test is often different (in 62 % of cases) from the gold-standard flush test. Third, although the mean difference between NIBP and IABP is usually small, and there is significant correlation between the two measurements, there is wide variability in the difference as evidenced by wide SD, IQR, and limits of agreement. Fourth, we identified no predictors of an optimally damped AL, suggesting that the AL mechanical setup is more important than patient-relevant variables. Finally, there were some predictors of a larger difference in NIBP–IABP; in particular, a vasodilator in use (where the NIBP overestimates DBP and MAP), and an optimally damped AL (where the NIBP underestimates the DBP and MAP). In addition, when inotropes are in use the NIBP may underestimate the SBP.

There are several implications of these findings for practice in the PICU. First, monitoring of IABP is often done with non-optimally damped AL, and how to address this problem should be a priority. We did not find any patient-relevant variables that predict this, and did not examine potential mechanical causes of this problem in this study (e.g., air bubbles, clots, excessive tubing, etc.). Nevertheless, this study brings attention to the problem, and suggests further study is needed to improve this situation. Second, when testing IABP accuracy, a flush test is required, as the stopcock test is not useful. Methods of flush testing in infants are currently being tested in our PICU. Third, there is clinically relevant variability in blood pressure measured by IABP and NIBP, even using optimally damped AL, and this applies to SBP, DBP, and MAP. Thus, in a patient suspected of having abnormal blood pressure, or on vasoactive infusions, the NIBP does not appear accurate enough to guide diagnosis and management.

To our knowledge, there has been little study of the accuracy of AL blood pressure monitoring in children. A recent study in adults undergoing major vascular and cardiac surgery found that 30.7 % of AL were under damped, resulting in clinically significant overestimation of SBP and MAP compared to NIBP [3]. In the optimally damped AL, the differences in BP were on average small, but with wide ranges and wide limits of agreement [3]. In the only pediatric study comparing NIBP and IABP (n = 40 children) it was found that NIBP “may seriously underestimate the severity of hypertension and hypotension in PICU patients potentially leading to undertreatment” [6]. However, in this study the damping category of AL was not determined [7], the clinical characteristics of the children were not described, and the variance of measurements was not reported. Several studies of adults in intensive care have identified wide variability in NIBP–IABP [3, 4, 8–10], although one study (in which flush tests were not performed) suggested that NIBP is accurate enough to detect MAP <65 mmHg [10]. Several studies in newborn babies (none reporting use of the flush test) also suggest significant variability between NIBP and IABP measurements predominantly using an umbilical AL [11–13]. One study in children using manual sphygmomanometry found the difference between this and optimally damped IABP was −1 (SD 12) for SBP and 7 (SD 12) for DBP; the patients’ clinical conditions were not reported [14]. The results of our study are broadly similar to these previous studies.

There are limitations to this study. This was a single-center study, and we do not know if the results can be generalized to other PICUs. Although we included 100 patients and 147 AL tests, the numbers of patients having the flush test (n = 66 and 29, respectively) and thus, with proven optimally damped AL (n = 30 and 13, respectively) are fairly small. We did not examine the mechanical setup of the AL or NIBP. The multiple statistical testing may have identified spurious predictors of differences in NIBP–IABP. Despite these limitations, the findings from this study are similar to those from a recent adult study [3], and from the only other pediatric study [6], suggesting generalizability of the findings. Although the numbers are modest, this is the only study reporting the accuracy of AL in PICU, and the largest study examining differences in NIBP–IABP in critically ill children. Although the examination of the mechanical setup was not included in the study protocol, the instructions did specify that prior to the AL tests the bedside nurse determined the AL was “zeroed; levelled; free of bubbles” and was “working for blood draws”, and the “NIBP cuff is optimal: bladder 40 % of arm circumference”. The study limitations do not change the main findings, that is, that AL in PICU are often not optimally damped, and that there are clinically relevant differences between NIBP and IABP even when using optimally damped AL. Further study should confirm these findings in other PICUs.

## Conclusions

In critically ill children, AL are often not optimally damped and thus, often give inaccurate measurements of IABP. The stopcock test is not useful to determine the damping condition of the AL, and a flush test is necessary. The NIBP may not be accurate enough to guide management compared to an optimally damped AL reading of IABP, particularly when the patient is on a vasodilator or inotrope infusion. These findings should be confirmed in a different PICU.

## Key messages

- Optimally damped arterial lines occur in about half of critically ill children, and this is not predictable by demographic or clinical variables
- There is much variability in ∆BP between NIBP and the gold standard IABP, and this varies even in the same patient on the same day, and is not easily predictable
- In critically ill children, NIBP may not be accurate enough to guide management, and more attention is needed to ensuring the arterial line is optimally damped

## Additional files

Additional file 1: The case report form for the study. (PDF 100 kb) Additional file 2: The study manual for the study. This file provides the definitions used, and the details of methods for determining the damping condition of the arterial lines. (PDF 406 kb) Additional file 3: Demonstration of setting up an arterial line (Part 1), zeroing and performing the flush test on an arterial line (Part 2), and calculations for determining the damping condition of the arterial line (Part 3). (WMV 29043 kb) Additional file 4: Supplemental figures. **Figure S1.** Bland Altman plots for non-invasive blood pressure compared to the invasive arterial blood pressure for all 147 arterial lines. **a** Systolic blood pressure difference; **b** diastolic blood pressure difference; **c** mean blood pressure difference. **Figure S2.** Difference between blood pressure measurements on the same day. **a** Systolic blood pressure difference; **b** diastolic blood pressure difference; **c** mean blood pressure difference. (PDF 133 kb)
