# Supplementary material for: The accuracy of blood pressure measured by arterial line and non-invasive cuff in critically ill children
Source: Crit Care. 2016 Jun 8;20:177. doi: 10.1186/s13054-016-1354-x (PMC4897864; doi:10.1186/s13054-016-1354-x)
Supplement: Additional file 1: — The case report form for the study. (PDF 100 kb) [file 13054_2016_1354_MOESM1_ESM.pdf]

The accuracy of blood pressure measured by arterial line and non-invasive cuff in critically ill children

ESM_1_Joffe:

The study case report form used for data collection.

Authors: Rachel Joffe BSc candidate[1], Jonathan Duff MD[2], Gonzalo Garcia Guerra MD, MSc[2], Jodie Pugh RN, Ari R Joffe MD[2]

Affiliations: 1. University of Alberta, Faculty of Science; 2. University of Alberta and Stollery Children's Hospital, Department of Pediatrics, Edmonton, Alberta, Canada.

Corresponding Author: Jonathan Duff MD; 4-546 Edmonton Clinic Health Academy; 11405 87 Avenue; Edmonton, Alberta, Canada; T6G 1C9; Phone: 780 2485435; Email: jon.duff@ahs.ca

BLOOD PRESSURE MEASUREMENT IN PICU

FORM 1. INCLUSION/EXCLUSION CRITERIA

1.1 INCLUSION CRITERIA: (all inclusion criteria must be answered "YES" to include patient)

YES	NO	
___	___	1. Admitted to the PICU at Stollery Children's Hospital
___	___	2. Arterial line in situ
___	___	3. Arterial line working for blood draws

1.2 EXCLUSION CRITERIA (all exclusion criteria must be answered "NO" to include patient)

YES	NO	
___	___	1. Unable to perform NIBP measurement
___	___	2. Venoarterial ECMO or LVAD
___	___	3. Subclavian flap used for repair of aortic arch in NIBP arm (e.g. coarctation repair, interrupted aortic arch repair)

1.3 DAY OF ARTERIAL LINE

|__| Day 1-3 (day category 1)
|__| Day 4-6 (day category 2)
|__| Day 7-10 (day category 3)

Date of arterial line insertion: ________________________

Specific day of arterial line: ___________

Patient ID Number |_|_|_| - Day Category |_|

BLOOD PRESSURE MEASUREMENT IN PICU

FORM 2. DEMOGRAPHICS

**2-1.**	**Age:**	|__|__| years	
		|__|__| months	
	If < 1 month	|__|__| days	
**2-2.**	**Gender:**	|__| *Male* |__| *Female*	
**2-3.**	**Diagnostic Category**	|__| Post-operative CV Surgery	
		|__| Non-operative Heart Disease	
		|__| Post-operative (non-CV)	
		|__| Shock (Medical)	
		|__| Respiratory	
		|__| Gastrointestinal	
		|__| Neurologic (Medical)	
		|__| Trauma	
		|__| Other: ____________________	
**2-4.**	**Inotrope scores**	**At time of BP measurement #1**	**#2**
	**Dopamine (mcg/kg/min)**	|__|__.__|	|__|__.__|
	**Dobutamine (mcg/kg/min)**	|__|__.__|	|__|__.__|
	**Epinephrine [Epi] (mcg/kg/min)**	|__.__|__|	|__.__|__|
	**Norepinephrine [NorEpi] (mcg/kg/min)**	|__.__|__|	|__.__|__|
	**Milrinone (mcg/kg/min)**	|__.__|__|	|__.__|__|
	**Vasopressin (U/kg/min)**	|__.__|	|__.__|
	**TOTAL INOTROPE SCORE**	|__|__|__|	|__|__|__|
**2-5.**	**Vasodilators [also includes milrinone]**	|__| *Yes* |__| *No*	
	**Nitroprusside [nipride] (mcg/kg/min)**	|__.__|__|	|__.__|__|
	**Nitroglycerin (mcg/kg/min)**	|__.__|__|	|__.__|__|
**2-5.**	**Ventilation**	|__|Invasive	|__|Invasive
		|__|Non-invasive	|__|Non-In
		|__|High-flow	|__|High-F
		|__|Not ventilated	|__|Not V

BLOOD PRESSURE MEASUREMENT IN PICU

OTHER DEMOGRAPHICS

	YES	NO
**Obesity:**	__	__
**Weight: _______kg**	**Height: __________ cm**	
**Severe Edema:**	__	__
**Chronic Hypertension:**	__	__
**Obstructive airway disease:**	__	__

BLOOD PRESSURE MEASUREMENT IN PICU

FORM 3. BLOOD PRESSURE MEASUREMENTS

3-1. Arterial Line

			Left or Right
Peripheral Site:	|__|	Radial	
	|__|	Ulnar	|__| 24 gauge (yellow)
	|__|	Brachial	|__| 22 gauge (blue)
	|__|	Posterior tibial	|__| 20 gauge (pink)
	|__|	Dorsalis pedis	
Central Site:	|__|	Femoral	|__| 2.5 French
			|__| 3.0 French
			|__| Larger (e.g. a sheath)

3-2. Flush test (>10kg)

**Performed:**	|__| *Yes* |__| *No*	|__| *Yes* |__| *No*
**Printed:**	|__| *Yes* |__| *No*	|__| *Yes* |__| *No*
**Natural Frequency:**	_____Hz |__| *No ringing*	_____Hz |__| *No ringing*
**Amplitude Ratio:**	_____ |__| *No ringing*	_____ |__| *No ringing*
**Damping:**	|__| *Optimal*	|__| *Optimal*
	|__| *Overdamped*	|__| *Overdamped*
	|__| *Underdamped*	|__| *Underdamped*
**Stopcock test (all)**		
**Natural Frequency:**	_____Hz |__| *No ringing*	_____Hz |__| *No ringing*
**Amplitude Ratio:**	_____ |__| *No ringing*	_____ |__| *No ringing*
**Damping:**	|__| *Optimal*	|__| *Optimal*
	|__| *Overdamped*	|__| *Overdamped*
	|__| *Underdamped*	|__| *Underdamped*
**Same as for flush test**	|__| *Yes* |__| *No*	|__| *Yes* |__| *No*

3-3 Blood pressure measurement

**Date Measured:**	|__|__| |__|__| |__|__|__|__| *DD /M M /Y E A R*	
**Interval (hours)**		|__|__|
**Time Measured:**	|__|__| : |__|__|	|__|__| : |__|__|
**Invasive Blood Pressure:**	|__|__|__|/|__|__|__| Mean: |__|__|__|	|__|__|__|/|__|__|__| Mean: |__|__|__|
**NIBP:**	|__|__|__|/|__|__|__| Mean: |__|__|__|	|__|__|__|/|__|__|__| Mean: |__|__|__|
**HR:**	|__|__|__|	|__|__|__|

BLOOD PRESSURE MEASUREMENT IN PICU

If repeated:

**Invasive Blood Pressure:**	|__|__|__|/|__|__|__| Mean: |__|__|__|	|__|__|__|/|__|__|__| Mean: |__|__|__|
**NIBP:**	|__|__|__|/|__|__|__| Mean: |__|__|__|	|__|__|__|/|__|__|__| Mean: |__|__|__|
**HR:**	|__|__|__|	|__|__|__|

Location of BP Cuff during first measurement :

|__| Opposite arm as arterial line
|__| Same arm as arterial line
|__| Leg on opposite side as arterial line
|__| Leg on same side as arterial line

Location of BP Cuff during second measurement:

|__| Opposite arm as arterial line
|__| Same arm as arterial line
|__| Leg on opposite side as arterial line
|__| Leg on same side as arterial line

FORM 4. SIGN OFF SHEET

Case Report Form to be signed off when the data has been checked as accurate and complete.

Research Assistant: ______________________________ **Date:** ______________________

Study Investigator: ______________________________ **Date:** ______________________
