# Supplementary material for: The accuracy of blood pressure measured by arterial line and non-invasive cuff in critically ill children
Source: Crit Care. 2016 Jun 8;20:177. doi: 10.1186/s13054-016-1354-x (PMC4897864; doi:10.1186/s13054-016-1354-x)
Supplement: Additional file 2: — The study manual for the study. This file provides the definitions used, and the details of methods for determining the damping condition of the arterial lines. (PDF 406 kb) [file 13054_2016_1354_MOESM2_ESM.pdf]

The accuracy of blood pressure measured by arterial line and non-invasive cuff in critically ill children

ESM_2_Joffe:

The study case report form manual used for data collection.

Authors: Rachel Joffe BSc candidate[1], Jonathan Duff MD[2], Gonzalo Garcia Guerra MD, MSc[2], Jodie Pugh RN, Ari R Joffe MD[2]

Affiliations: 1. University of Alberta, Faculty of Science; 2. University of Alberta and Stollery Children's Hospital, Department of Pediatrics, Edmonton, Alberta, Canada.

Corresponding Author: Jonathan Duff MD; 4-546 Edmonton Clinic Health Academy; 11405 87 Avenue; Edmonton, Alberta, Canada; T6G 1C9; Phone: 780 2485435; Email: jon.duff@ahs.ca

Form 1 – Inclusion/Exclusion Criteria:

1.1 Inclusion Criteria:

Check "**yes**" for each inclusion criteria present.
NOTE: all inclusion criteria must be checked "yes" in order for the patient to be eligible for the study.

1.2 Exclusion Criteria:

Check "**no**" for each exclusion criteria NOT present.
NOTE: all exclusion criteria must be checked "no" in order for the patient to be eligible for the study.

1.3 Day of Arterial Line:

We will record an IBP-NIBP pair in each patient up to twice on the same day {only in day 1-3 category), at least 2 hours apart.

Use a separate form for each patient on day 1-3 of the art line; day 4-6 of the art line; and day 7-10 of the art line. Since these days will be analyzed separately by the day category, each patient can contribute up to **only once to each** of the three day categories (i.e. on day 1 or 2 or 3, not on more than one; on day 4, 5, or 6, but not on more than one of these).

Day 1 is the day of arterial line (i.e., day of insertion of arterial line)

<u>Form 2 – Demographics:</u>

Age: Enter the patient's age

Gender: Check either male or female

Diagnostic Category (primary admitting diagnosis):

Post-operative CV surgery:	Patient recovering from repair of a congenital or acquired heart lesion (includes sternotomy/thoracotomy)
Non-operative heart disease:	Patient with congenital or acquired (e.g. myocarditis) heart disease prior to any operative intervention, or not having any operative intervention this admission
Post-operative (non-CV)	Patient recovering from surgical procedure (non-cardiac) such as general surgery, neurosurgery, or ENT
Shock (Medical):	Septic shock, hypovolemic shock (dehydration, bleeding), vasodilatory shock (anaphylaxis, overdose, spinal trauma),
Respiratory:	Respiratory distress/failure (pneumonia, bronchiolitis, aspiration, acute respiratory distress syndrome)
Gastrointestinal:	Gastrointestinal bleeds, liver failure/transplant
Neurologic (Medical):	Coma, seizures
Trauma:	Traumatic brain injury, liver or spleen laceration, multitrauma
Other	If not captured in above, write admitting diagnosis

Inotrope Score:

Dopamine dose (μg/kg/min) + dobutamine dose (μg/kg/min) + 100 x epinephrine dose(μg/kg/min) + 100 x norepinephrine dose (μg/kg/min) + 10 x milrinone dose(μg/kg/min) + 10,000 x vasopressin dose (U/kg/min)

*** Note vasopressin can be run U/kg/**hr** as well ***

Vasodilators: Nitroprusside, nitroglycerin

Ventilation mode:

Invasive	Ventilation through an endotracheal/tracheostomy tube
Non-invasive	Ventilation through a mask (nasal or full face) interface (CPAP, BiPAP)
High-flow	Ventilation through high-flow/OptiFlow nasal cannula
Not ventilated	None of the above

<u>Other Demographics:</u>

1. Obesity:

-weight greater than 95% percentile for age on the growth chart

2. Severe edema on the arm where cuff pressure is being done:

-defined as noticeable severe edema as stated by the bedside nurse

3. Chronic Hypertension:

-known to have a diagnosis of hypertension prior to admission to PICU, and on medication for this at baseline.

4. Obstructive airway disease:

-this is any of the following: asthma, bronchiolitis, cystic fibrosis.

<u>Form 3 – Blood Pressure Measurements</u>

Arterial Line Site: Choose the source of the invasive arterial line measurement

Peripheral Sites	Radial	Wrist (thumb side)
	Ulnar	Wrist (pinky side)
	Brachial	Elbow
	Posterior Tibial	Behind the ankle
	Dorsalis Pedis	Top of the foot

Enter the size of the catheter: 24 (yellow), 22 (blue) or 20 (pink)

Central Site	Femoral	Groin

Enter the size of the catheter: Measured in French (2.5 or 3.0 or larger)

Flush Test: Prior to each invasive BP measurement, ensure arterial line was immediately flushed, and record the arterial wave trace during and for at least 3 beats after the flush. This can be done in all patients at least 10kg of weight.

Stopcock Test: By turning the stopcock off for a few seconds, then opening it, this will provide another version of the flush test. This is the only way to do the flush test on those under 10kg of weight.

Invasive BP: Record the invasive BP (systolic/diastolic and mean) during the NIBP cuff deflation just, with date and time. The optimal time is when the NIBP pressure appears on the monitor screen.

NIBP: Record non-invasive BP measurement. If systolic BP discordant by more than 10 mmHg from IBP, then repeat the NIBP and IBP and record both sets on the CRF. **Enter into the database only the most concordant (using systolic BP) of the two measurements.**

HR: The heart rate on the monitor during deflation of the NIBP cuff.

Details of the Flush Test:

-using an Intraflo continuous flush element, or a quick turn of the stopcock.

-check that the arterial line is zeroed; leveled; free of bubbles (flushed).

-check that the NIBP cuff is optimal: bladder 40% of arm circumference and 80% length of upper arm (too small results in falsely high readings).

1. **Calculate the Natural Frequency**: how rapidly the system oscillates after a stimulus
2. **Calculate the Amplitude Ratio**: how quickly the system comes to rest after a stimulus (frictional forces)
3. **If no ringing**: cannot calculate the above values, then the system is overdamped.

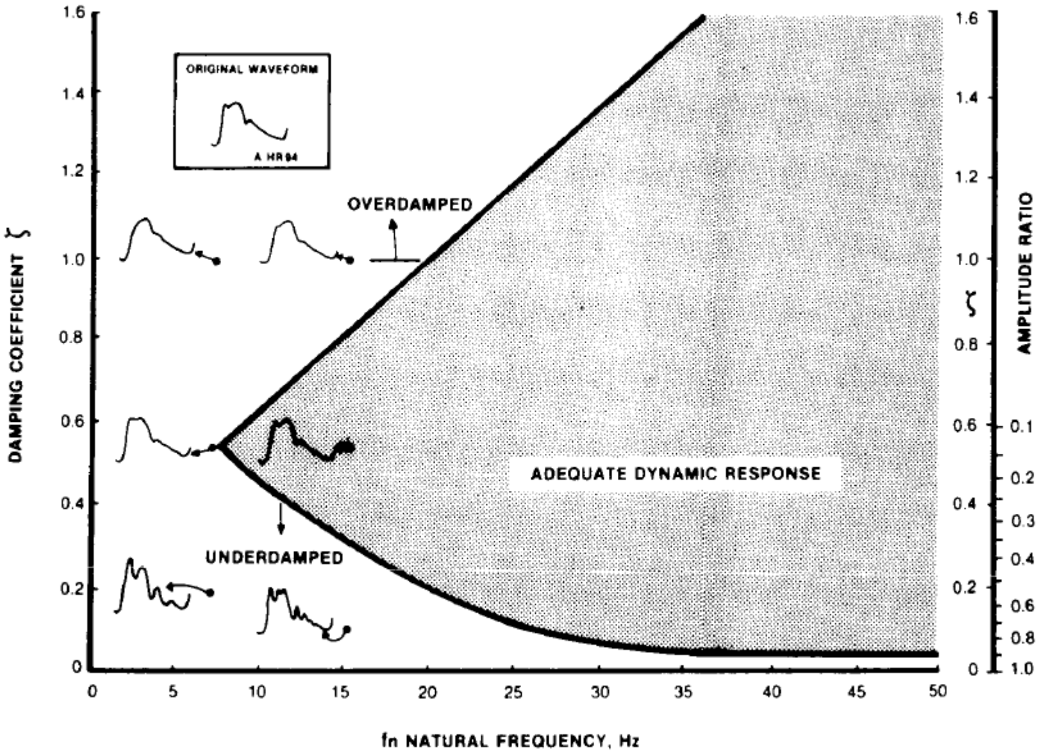

1.0 is 0

0.80 is 0.02

0.70 is 0.05

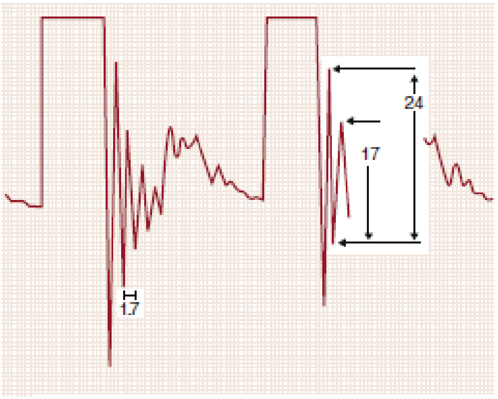

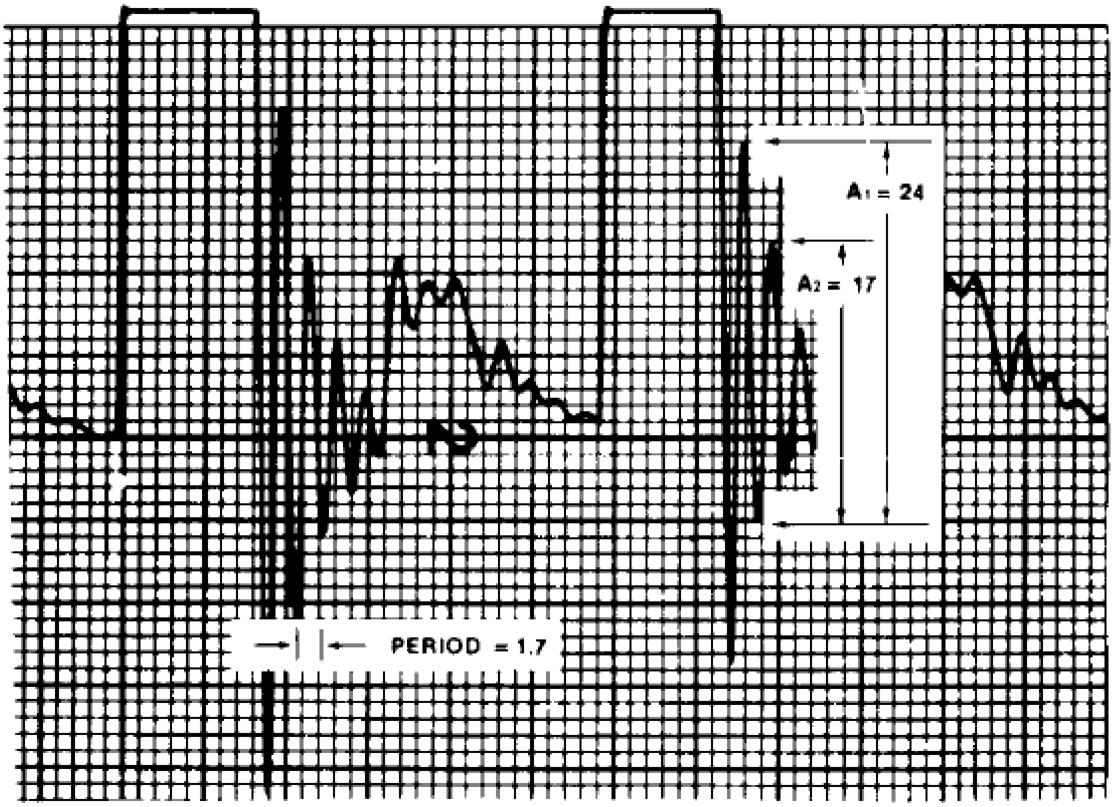

Natural frequency = paper speed mm/sec ÷ one cycle measured in mm

-in this example, the natural frequency is 25/1.7 = 15Hz

-if too low, frequencies measured in the monitored pressure waveform will overlap that of the measurement system, the system will resonate, and pressure waveforms recorded on the monitor will be exaggerated. Optimized (higher) if short length stiff tubing, limited stopcocks, no clots/bubbles

Amplitude ratio = amplitude of second peak ÷ amplitude of first peak.

-Note, this could be the amplitude ratio of any two successive peaks, not necessarily the first and second.

-in this example, the amplitude ratio = 17/24 = 0.71

-this can be converted to a damping coefficient.

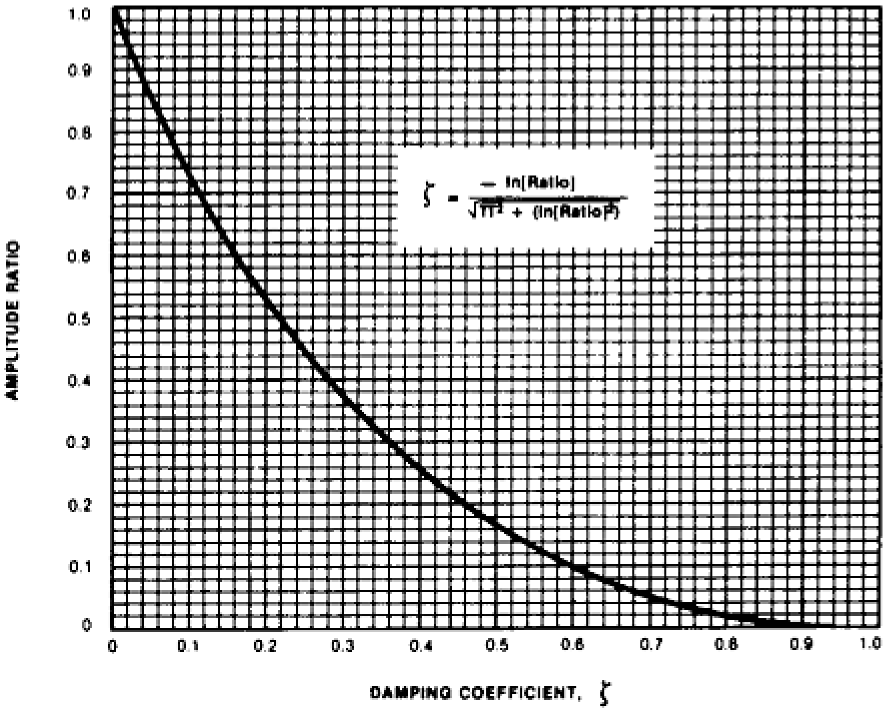

NOTE: two (or three) flushes may be required to see these successive peaks. This is because if the flush ends during systole, the superimposed systolic peak might make it difficult to see.

Damping: anything that reduces energy in an oscillating system.

-Usually, optimum is when a flush results in one undershoot followed by a small overshoot, then settles to the patient's waveform.

-Overdamped: slurred upstroke, absent dicrotic notch, loss of fine detail, falsely narrow pulse pressure, lower SBP, higher DBP.

-Underdamped: systolic pressure overshoot, additional small nonphysiologic pressure waves distorting waveform (hard to discern dicrotic notch). Due to the tendency of a system to oscillate with greater amplitude at some frequencies than at others.

Examples:

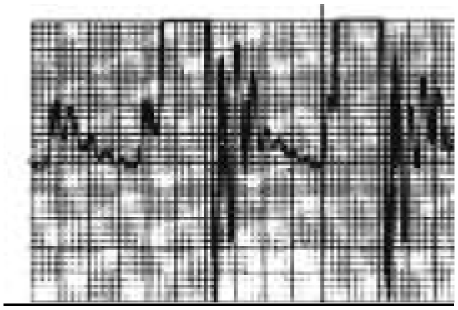

Underdamped: Overshoot (increase in SBP) and ringing (oscillation). Distorted by the systolic pulsation of the aortic pressure signal.

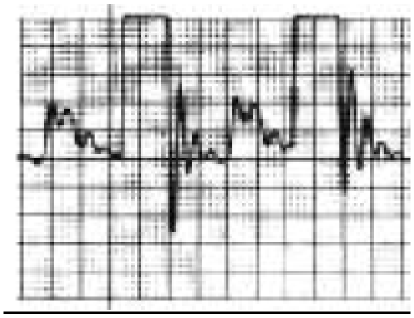

Underdamped: but less overshoot and less ringing.

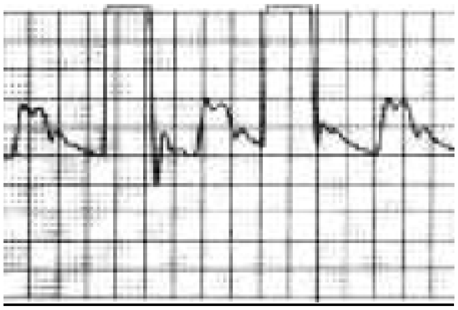

Near optimal: little ringing

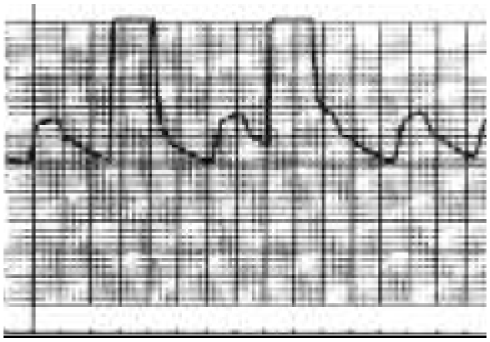

Overdamped: cannot easily determined natural frequency or amplitude ratio because there is no ringing.

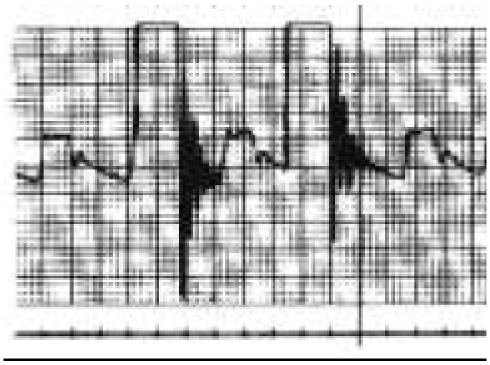

Higher natural frequency. Still underdamped.

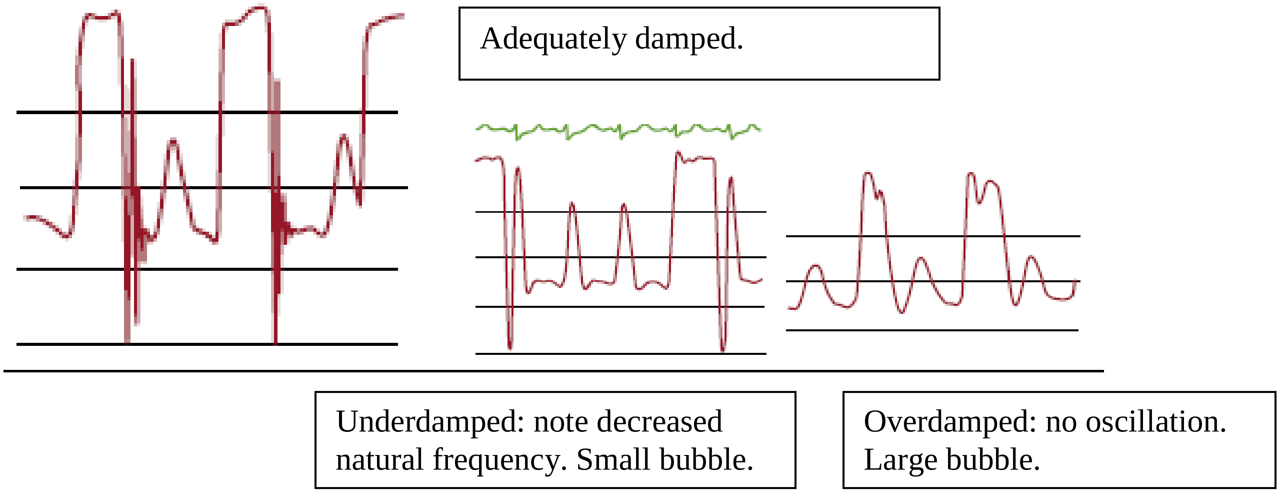

References: figures are taken from the following-

1. Gardner RM: **Direct blood pressure measurement- dynamic response requirements.** *Anesthesiology*, 1981;**54:**227-236.

2. Schroeder B, Barbeito A, Bar-Yosef S, Mark JB: **Chapter 45: Cardiovascular monitoring.** In *Miller's Anesthesia 8th Edition*. Edited by Miller RD. Saunders: USA; 2014:1345-1395.
