# Supplementary material for: The accuracy of blood pressure measured by arterial line and non-invasive cuff in critically ill children
Source: Crit Care. 2016 Jun 8;20:177. doi: 10.1186/s13054-016-1354-x (PMC4897864; doi:10.1186/s13054-016-1354-x)

The accuracy of blood pressure measured by arterial line and non-invasive cuff in critically ill children

Additional File 3.Joffe (pdf): Supplemental figures.

Figure E1. Bland Altman plots for non-invasive blood pressure compared to the invasive arterial blood pressure for all 147 arterial lines. a. Systolic blood pressure difference; b. diastolic blood pressure difference; c. mean blood pressure difference.

Figure E2. Difference between blood pressure measurements on the same day. a. Systolic blood pressure difference; b. diastolic blood pressure difference; c. mean blood pressure difference.

The study case report form used for data collection.

Authors: Rachel Joffe BSc candidate[1], Jonathan Duff MD[2], Gonzalo Garcia Guerra MD, MSc[2], Jodie Pugh RN, Ari R Joffe MD[2]

Affiliations: 1. University of Alberta, Faculty of Science; 2. University of Alberta and Stollery Children's Hospital, Department of Pediatrics, Edmonton, Alberta, Canada.

Corresponding Author: Jonathan Duff MD; 4-546 Edmonton Clinic Health Academy; 11405 87 Avenue; Edmonton, Alberta, Canada; T6G 1C9; Phone: 780 2485435; Email: jon.duff@ahs.ca

Figure E1. Bland Altman plots for non-invasive blood pressure compared to the invasive arterial blood pressure for all 147 arterial lines. a. Systolic blood pressure difference; b. diastolic blood pressure difference; c. mean blood pressure difference.

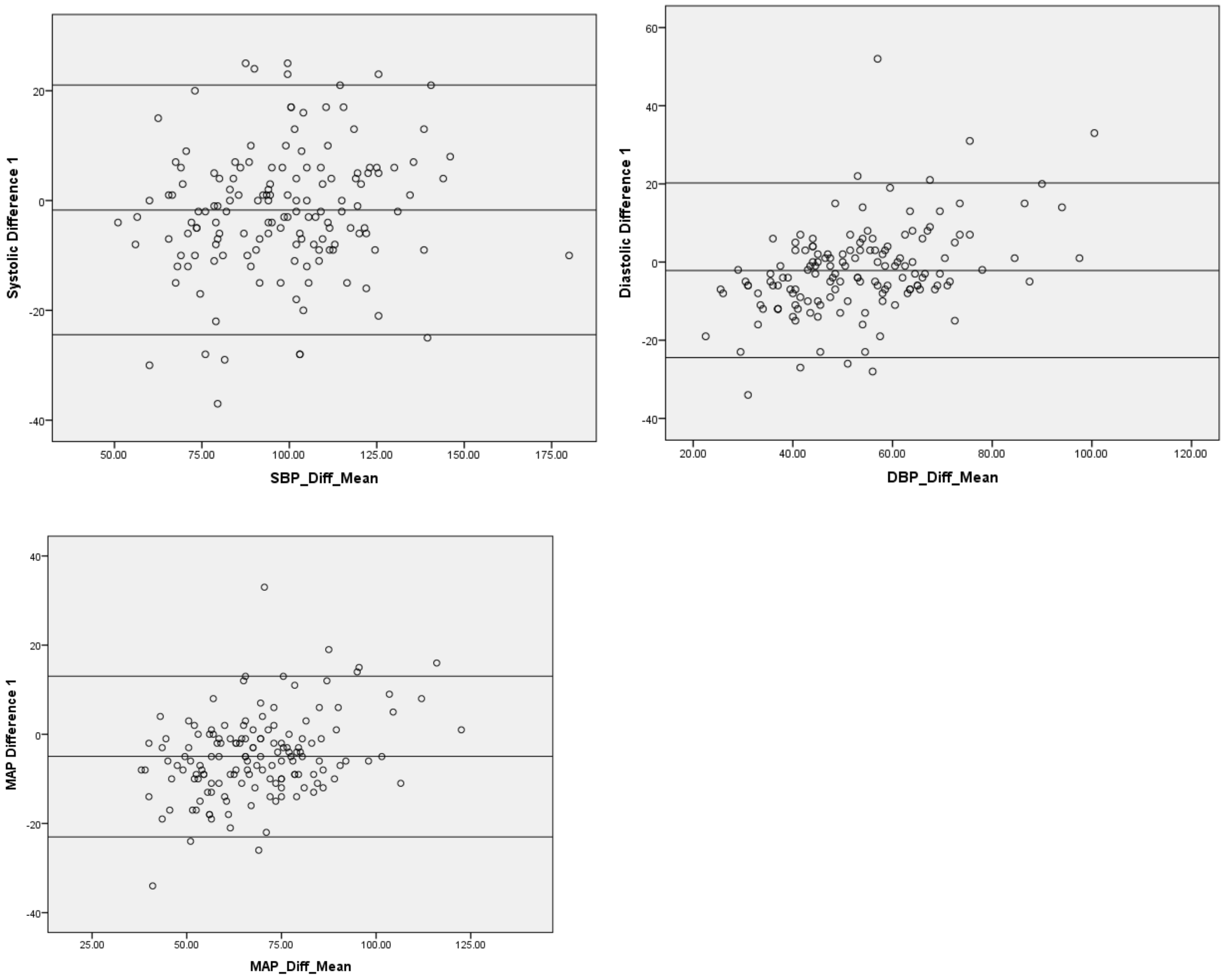

Figure E2. Difference between blood pressure measurements on the same day. a. Systolic blood pressure difference; b. diastolic blood pressure difference; c. mean blood pressure difference.

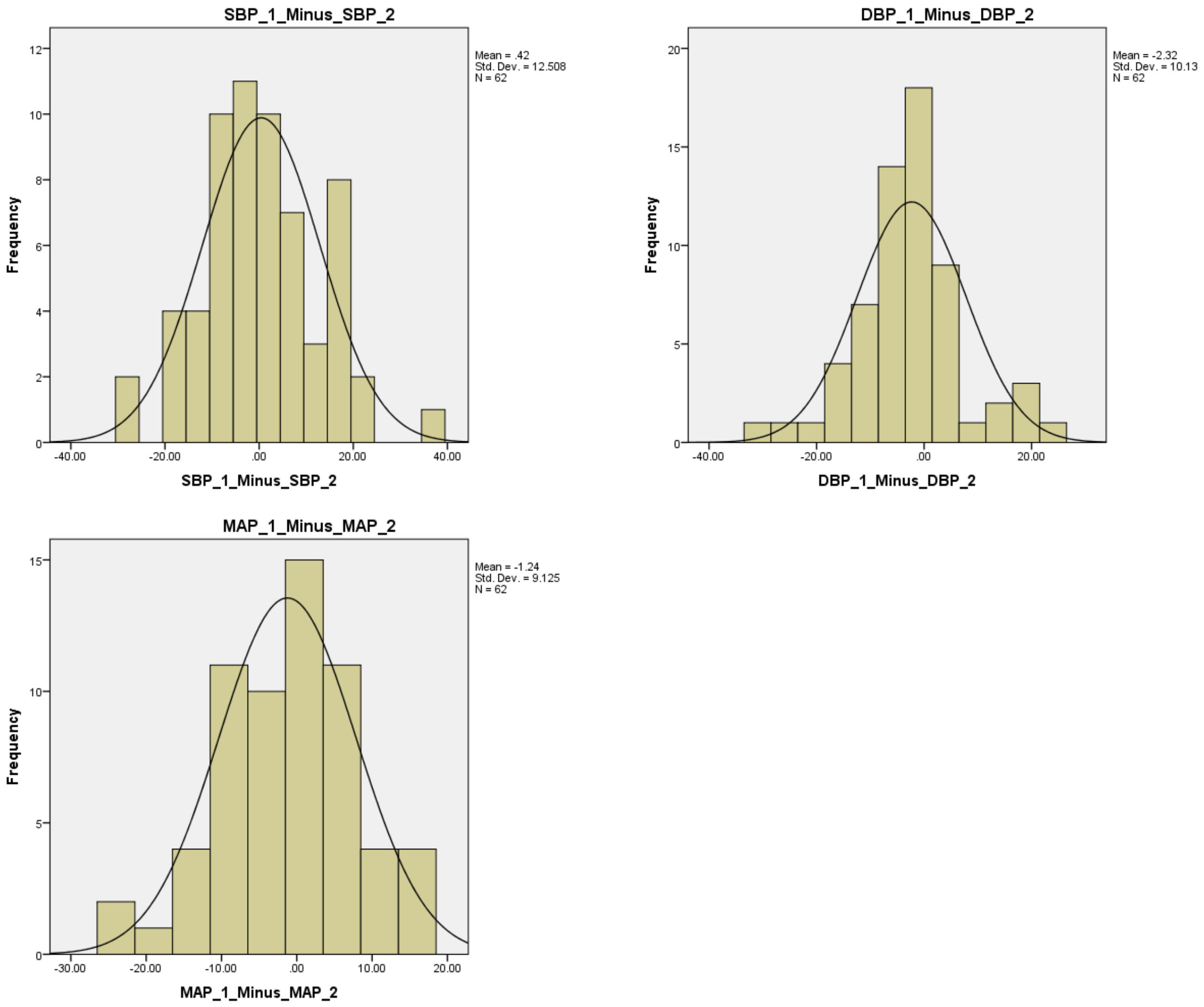

Supplement: Additional file 4: — Supplemental figures. Figure S1. Bland Altman plots for non-invasive blood pressure compared to the invasive arterial blood pressure for all 147 arterial lines. a Systolic blood pressure difference; b diastolic blood pressure difference; c mean blood pressure difference. Figure S2. Difference between blood pressure measurements on the same day. a Systolic blood pressure difference; b diastolic blood pressure difference; c mean blood pressure difference. (PDF 133 kb) [file 13054_2016_1354_MOESM4_ESM.pdf]
